# Antioxidant Activity of Anthocyanins and Anthocyanidins: A Critical Review

**DOI:** 10.3390/ijms252212001

**Published:** 2024-11-08

**Authors:** Izabela Sadowska-Bartosz, Grzegorz Bartosz

**Affiliations:** Laboratory of Analytical Biochemistry, Institute of Food Technology and Nutrition, College of Natural Sciences, Rzeszow University, 4 Zelwerowicza Street, 35-601 Rzeszow, Poland; gbartosz@ur.edu.pl

**Keywords:** antioxidant, antioxidant assays, indirect antioxidant effect, oxidative stress, reactive oxygen species, biomarkers

## Abstract

Anthocyanins are the main plant pigments responsible for the color of flowers, fruits, and vegetative organs of many plants, and are applied also as safe food colorants. They are efficient antioxidants. In this review, the reactivity of anthocyanins and their aglycones, anthocyanidins, in the main antioxidant assays, and their reactions with reactive oxygen and nitrogen species, effects of interactions with other compounds and metal ions on the antioxidant activity of anthocyanins and the electrochemical properties of anthocyanins are presented. Numerous cases of attenuation of oxidative stress at the cellular and organismal levels by anthocyanins are cited. The direct and indirect antioxidant action of anthocyanins, the question of the specificity of anthocyanin action in complex extracts, as well as limitations of cellular in vitro assays and biomarkers used for the detection of antioxidant effects of anthocyanins, are critically discussed.

## 1. Introduction

Anthocyanins (from the Greek *anthos*, flower and *kianos*, blue), a class of flavonoids, are the most important pigments of vascular plants. They are responsible for the shiny orange, pink, red, violet, and blue colors of flowers and fruits, but also of vegetative organs of many plants. Anthocyanins have many diverse functions within plants. Synthesis of anthocyanins in petals is intended to attract pollinators, whereas anthocyanins in seeds and fruits may aid in seed dispersal. Anthocyanins and other flavonoids can also be important as feeding deterrents and in the protection against damage from UV irradiation and other stresses [1,2,3,4]. Anthocyanins and their derivatives are the crucial pigments responsible for the red wine color [5,6]. As all flavonoids, they are efficient antioxidants and exert many beneficial health effects, which may depend on or be independent of, their antioxidant action [7,8,9]. They are used as safe food colorants, with no adverse effects on consumers, in contrast to many synthetic pigments [10,11].

The best nutritional sources of anthocyanins are berries such as strawberries, blueberries, blackberries, blackcurrants, redcurrants, and raspberries. The highest anthocyanin content is found in elderberries and chokeberries. Other good sources of anthocyanins include purple corn, cherries, plums, pomegranates, eggplants, wine, grapes, and red/purple vegetables such as black carrots, red cabbage, purple cauliflower, maqui, myrtle, and açai [12,13,14,15,16].

**Figure 1:**
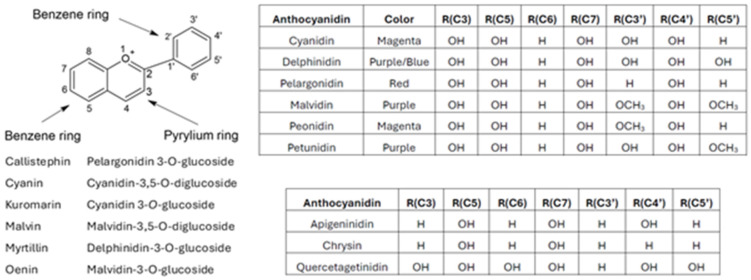
Numbering of anthocyanidin carbon atoms, structures of the most common anthocyanidins and other anthocyanidins discussed in the article, and vocabulary of common anthocyanins.

Anthocyanins present in plants are *O*-glycosides of aglycons (anthocyanidins) and sugars. Anthocyanidins differ in substituents on the flavylium (2-phenylbenzopyrylium) skeleton. From among 31 identified anthocyanidins present in about 700 anthocyanins, six are the most common (Figure 1).

Anthocyanins have a sugar residue linked to an anthocyanidin in position C3 of the aglycone, via an α- or β-glycosidic bond. If additional sugar residues are present in the anthocyanin molecule, they are linked in positions C5 and C7, and less frequently in positions C3′ and C5′. The sugar residues are usually glucose, galactose, rhamnose, arabinose, or xylose. Anthocyanin molecules can be additionally acylated with cinnamic acids (especially cinnamic or caffeic acid, but also ferulic or sinapic acid), acetic, malic, malonic, oxalic, or succinic acid [17,18,19]. Glycosylation increases the stability and water solubility of anthocyanidins. Increasing the number of sugar residues seems to further increase the stability of the anthocyanin, which is explained by the higher stability of the network of intramolecular H-bonds within the sugar moiety, and between the sugar and the aglycone [20,21]. Anthocyanin glucosyl acylation by aliphatic and aromatic acids further increases their stability [22].

**Figure 2:**
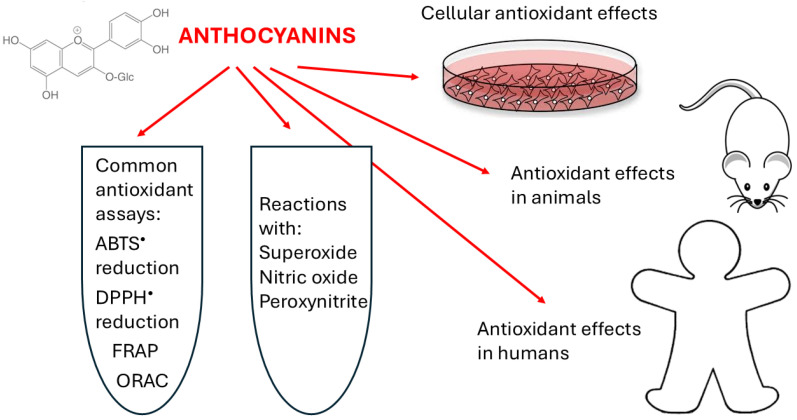
Antioxidant activity of anthocyanins discussed in this review.

Similar to other flavonoids, anthocyanins and anthocyanidins are good antioxidants, due to the scavenging of free radicals and other reactive oxygen and nitrogen species (ROS and RNS, respectively). This review summarizes the antioxidant activity of anthocyanins in cell-free systems, exemplifies their antioxidant effects on cells in vitro, in animal experiments, and in human studies (Figure 2), and discusses fields of uncertainty associated with these studies.

## 2. Anthocyanidins and Anthocyanins in the Popular Assays of Antioxidant Activity

In general, the antioxidant activity of anthocyanins (as well as other antioxidants) is due to two mechanisms: hydrogen atom transfer (HAT) and single-electron transfer (SET). In the HAT mechanism, a free radical R^•^ removes a hydrogen atom from the antioxidant. In this reaction, the free radical is converted into a more stable product and a free radical of the antioxidant A^•^ is formed. In the SET mechanism, the antioxidant donates an electron to the free radical, also forming A^•^. These two mechanisms often operate simultaneously, and their contribution is determined by the antioxidant structure, solubility, the partition coefficients of the reactants, and the solvent polarity [23]. From among the most popular assays of antioxidant activity, the Oxygen Radical Absorbing Capacity (ORAC) assay [24,25] is a HAT-based assay of antioxidant activity, while the Ferric Reducing Antioxidant Power (FRAP) assay [26] and CUPric ion Reducing Antioxidant C apacity (CUPRAC) [27] are SET-based assays. 2,2 DiPhenyl-1-PicrylHydrazyl radical (DPPH^•^) decolorization [28] and 2,2′-Azinobis(3-ethyl-2,3-dihydroBenzoThiazole-6-sulphonate) radical (ABTS^•^) decolorization assays [29] may involve both SET and HAT reactions [30].

Apart from the activity in the above mentioned reductive assays, of which DPPH^•^ and ABTS^•^ decolorization assays are based on reactions with non-physiological substrates and allow for only a general screening of the antioxidant capacity, of limited physiological relevance, anthocyanins were demonstrated to react with peroxyl radicals (such as radicals, though also non-physiological, like those employed in the ORAC assays and radicals propagating lipid peroxidation) and other reactive oxygen species such as superoxide radical anions [31,32], nitric oxide [33], hydrogen peroxide [34,35,36], and peroxynitrite [32,36,37].

Two properties are responsible for effective antiradical activity of a compound: (i) high reactivity with free radicals and (ii) high stability of its radical formed in these reactions, preventing its participation in chain free radical processes. For flavonoids, the following structural features, often referred to as “Bors criteria”, were proposed to condition the optimal antioxidant function: (i) the presence of a catechol group on the B ring that, due to hydrogen bonding, leads to high stability of the flavonoid radical formed, (ii) a 2,3 double bond in combination with a C4-oxo group on the C-ring, which facilitates electron delocalization, and (iii) the presence of OH groups in positions C3 and C5 in combination with the C4-oxo group that enables electron delocalization via hydrogen bonds [38,39].

The behavior of a range of flavonoids in the DPPH^•^ decolorization assay is dependent on the Bors criteria [40], but their reactivity in various antioxidant assays is only partially concordant with the predictions of Bors criteria [40,41,42]. The application of the Bors criteria to anthocyanins is limited as they lack the C4-oxo group; nevertheless, some of the Bors predictions seem valid.

The antioxidant activity of anthocyanins depends, first of all, on their aglycones. In reactions with oxidants, anthocyanidins are transformed into aroxyl radicals, which are comparatively more stable due to resonance, than the free radical that it has reduced. The overall result may include the termination of damaging oxidative chain reactions [43].

The antioxidant and antiradical activity of anthocyanidins and anthocyanins are strongly related to their structural features, including the kind, number, and position of substituents on the flavylium ion. The number and position of hydroxyl and methoxy substituents, as electron donating groups, were found to have a great impact on the antioxidant activity [44]. Two structural groups are important determinants of the radical scavenging activity of anthocyanidins: first, the ortho-dihydroxy structure in the B-ring; second, and the C1=C2 and C3=C4 double bonds in conjugation. In anthocyanidins with catechol (1,2-diphenol) or pyrogallol (1,2,3-triphenol) structures, the oxidation is carried out via free radicals until a stable semiquinone is formed. These anthocyanidins are especially easy to oxidize because the phenoxyl radical is quite stable and does not easily extract hydrogen from other substances, lasting enough time to react with another semiquinone; then, disproportionation generates a quinone and reconstitutes a phenol from the two interacting radicals. It is expected that anthocyanidins with a pyrogallol structure (delphinidin) or an *o*-dihydroxyl substitution (cyanidin and petunidin) will be the most active as antioxidants and thus more susceptible to oxidation, in agreement with Bors condition (i). In the case of pelargonidin, peonidin, and malvidin, which are not *o*-dihydroxyl substituted, oxidation is more difficult. From the six most common anthocyanidins, pelargonidin is expected to have the lowest antioxidant activity and, thus, is the most stable in neutral pH conditions [45]. The stability of semiquinone radicals is enhanced by extension of the radical delocalization over anthocyanidin rings B and C, and the two hydroxyl oxygen atoms [46,47]. The resulting radicals can also be stabilized by hydrogen bonding with neighboring hydroxyl groups [48,49]. The C1=C4 and the C1=C2 conjugation, as well as the C3=C5=C7 and C3′ and C4′ substituents on rings C, A, and B, respectively, are essential for the formation of different electronic delocalized and oxidized structures [50,51].

Glycosylation of anthocyanidins usually lowers their antioxidant activities (as the aglycones may be sterically hindered by the bulky sugar moieties) but may also enhance this activity depending on the type of anthocyanidin and sugar, and the oxidant concerned [50,52]. The site of attachment and number of glycosyl residues may affect the antioxidant capacity of anthocyanins [22]. For example, 3-glucosylation can either increase (petunidin and pelargonidin), not affect (malvidin and cyanidin), or decrease (delphinidin and petunidin) the antioxidant activity. The type of sugar showed different effects on anthocyanin activity; while no significant difference in activity was observed between cyanidin-3-glycosides with glucose or galactose, cyanidin-3-arabinoside showed significantly lower activity [50]. Oenin was found to have lower antioxidant activity than malvidin in the ABTS^•^ decolorization assay [53]. 3-*O*-glucosides of peonidin an petunidin were somewhat more effective in the DPPH^•^ scavenging assay than corresponding anthocyanidins, but delphinidin 3-*O*-glucoside (myrtillin) showed lower activity than delphinidin in this test. In protecting against 2,2-azobis (2-amidinopropane) (AAPH)-induced lipid peroxidation, 3-*O*-glucosides of peonidin, petunidin, and delphinidin were slightly more effective than corresponding anthocyanidins [54].

The higher activity of 3-*O*-glucoside anthocyanins than their aglycons could be due to the electron donating effect of the C3-bulky sugar group [55]. On the other hand, it was reported that diglucosylation of anthocyanidins at the C3 and C5 positions lowered the antioxidant activity [32,50]. Interestingly, cyanidin has a higher ORAC value than its anthocyanin [56]. An anthocyanin bearing an extra sugar at position C3, in the heterocyclic C-ring, has lower antioxidant activity than an anthocyanidin with a single sugar molecule [57]. Acylation of an anthocyanin with *p*-coumaric acid brought a significant increase in antioxidant activity, apparently due to the antioxidant activity of the coumaric acid residue itself. Apparently, for the same reason, delphinidin derivatives acylated with caffeic acid showed a higher antioxidant activity than their respective deacylated compounds [58]. Diacylation of the anthocyanin markedly increased the antioxidant activity but 5-glycosylation lead to a reduction in the activity [56].

**Figure 3:**
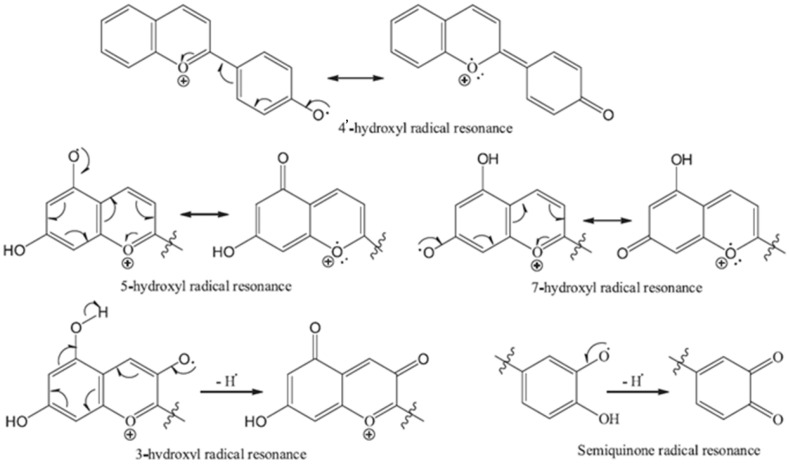
Stabilization resonance of various anthocyanidin radicals. Reproduced from [44], with permission.

Resonance within the anthocyanidin moiety is important for the stability of the radicals formed on anthocyanidins and anthocyanins, and affects the reactivity of these compounds, in agreement with Bors condition (iii). In one study, kuromanin had the highest ORAC activity among 14 anthocyanidins and anthocyanins, which was 3.5 times stronger than Trolox (a vitamin E analogue), while pelargonidin had the lowest antioxidant activity but was still as potent as Trolox [52]. Apigeninidin, having the C5 and C7 hydroxyl groups, had low reductive activity towards DPPH^•^ and low but much higher activity against ABTS^•^. The apigeninidin radical, upon dehydrogenation, can only have C5- or C7-OH radical resonance through the pyrylium oxygen (Figure 3). Chrysin has a similar structure to that of apigeninidin but with no C4′-OH group. Both anthocyanidins exhibited similar scavenging activities towards the ABTS^•^ [53], indicating similar stabilization effects of C4′-OH radical resonance and C5- or C7-OH radical resonance.

Most of the studied anthocyanidins and anthocyanins showed higher reducing activities than Trolox. The most active compounds towards all radicals were quercetagetinidin, delphinidin, petunidin, and their anthocyanins, while the least active compounds against all radicals were apigeninidin, pelargonidin, and pelargonidin-3-glucoside. In the FRAP assay, the anthocyanidins and anthocyanins studied, except for apigeninidin, showed good electron donating abilities, higher than Trolox [44]. The high activity of quercetagetinidin in the ABTS^•^ decolorization and FRAP assays can be attributed to the unusual presence of an additional hydroxyl group in the C6 position.

Pelargonidin has a similar structure to apigeninidin, but with an extra hydroxyl group in position C3; it showed significantly higher activities against all radicals. The higher activity can be attributed to the C3-OH radical resonance between C3-OH and C5- C7- or C4′-OH groups, and the formation of a stable diketonic product, which pushes the hydrogen transfer reaction forward. Radicals formed on C5-, C7-, or C4′-hydroxyl groups can also be stabilized by the same resonance in the opposite direction. This resonance effect is similar to that reported for the catechol structure, where stable semiquinone radicals are formed, leading to stable diketones [46,59]. The presence of the C3-OH group conjugated with a C2=C3 double bond was reported to enhance the antioxidant activity in other classes of flavonoids; this structural feature permits coplanarity of ring B with rings A and C, allowing extension of conjugation and electron delocalization, and leading to extra stability of the resulting radicals [60,61].

Cyanidin, which has an extra hydroxyl group in the C3′ position compared with pelargonidin, showed much higher scavenging activity. The higher activity of cyanidin is due to its catechol structure that allows for the well-known stabilization of a semiquinone radical and the formation of a stable quinone product. Petunidin showed more scavenging activity against all radicals than of cyanidin, since it bears a catechol structure but with one more methoxy group. The strongest reductants in the FRAP assay were delphinidin, petunidin, cyanidin, and their anthocyanins, plus quercetagetinidin, confirming that a catechol moiety enhances not only the radical scavenging activity but also the electron donating ability of anthocyanidins and anthocyanins, as mentioned above.

Delphinidin has more hydroxyl groups (additional -OH in the C5′ position) than cyanidin (pyrogallol structure) on ring B, which increases the scavenging activity towards all radicals studied. Quercetagetinidin has a pyrogallol moiety on ring A and a catechol moiety on ring B and, thus, also has a higher activity against the three radicals than cyanidin [44].

Another study showed the following reactivity sequence: cyanidin-3-glucoside ≈ cyanidin-3,5-diglucoside > delphinidin-3-glucoside > pelargonidin-3-glucoside in the DPPH^•^ decolorization assay, cyanidine-3-glucoside > cyanidin-3,5-diglucoside > pelargonidin-3-glucoside > delphinidin-3-glucoside in the ABTS^•^ decolorization assay, and cyanidin-3,5-diglucoside ≈ cyanidin-3-glucoside > delphinidin-3-glucoside > pelargonidin-3-glucoside in the FRAP assay [62]. In a next paper, the concentrations needed to scavenge 50% of DPPH^•^ in the assay (EC_50_ values) were 3.74, 4.85, 5.25, 7.29, and 10.9 µM for delphinidin, cyanidin, pelargonidin, kuromanin, and callistephin, respectively [63]. In a successive report, EC_50_ values for DPPH^•^ scavenging of kuromarin, petunidin 3-*O*-glucoside, myrtillin, oenin, and cyanidin 3-*O*-rutinoside were 6.61, 6.61, 6.90, 7.48, and 8.78 µM, respectively, lower than or comparable to that of ascorbic acid (7.28 µM) [64].

Structure-Activity Relationship (SAR) analysis points out that peonidin should have lower activity than cyanidin since it has the same structure with methylation of the C3′-hydroxyl group and lacks the catechol structure. On the other hand, it has higher activity than that of pelargonidin because of the extra C3′-OH group [59]; similar results were obtained by other authors [50]. Similar to peonidin, malvidin lacks a catechol structure but has one extra methoxy group in the C5′ position, due to which it has higher activity. The electronic effect of substituents has a large influence on both the activation energy and O–H bond dissociation energy of phenols [65]. It was proposed that methoxy and hydroxyl groups stabilize the resulted radicals of phenolic compounds, leading to an increase in their antioxidant activity, with a greater effect shown by the methoxy group than hydroxyl substituent [59,60,61]. Therefore, comparing the activity of compounds lacking the catechol structure, the activity against DPPH^•^ and ABTS^•^ decreases in the order of malvidin > peonidin > pelargonidin> apigeninidin which corresponds to the presence of 6, 5, 4, 3, and 1 hydroxyl and/or methoxy groups, respectively. The same order of activity (malvidin > peonidin > pelargonidin) was found experimentally in the ORAC test [52] and was predicted theoretically based on the calculation of O–H bond dissociation energies [66] and in the comparison of the reducing power measured by the FRAP assay, suggesting similar factors affecting both the reducing power and the radical scavenging activities of anthocyanidins and anthocyanins. It was observed that the reducing power increases with the number of hydroxyl and methoxy groups on ring B [47,61]. It was also observed that a compound which lacks a catechol structure, e.g., malvidin with four hydroxyl and methoxy groups, may have scavenging activities similar to, or even better than, that of a compound possessing a catechol structure, e.g., cyanidin with less stabilizing substituents.

## 3. Reactions of Anthocyanidins and Anthocyanins with Reactive Oxygen and Nitrogen Species

Anthocyanidins and anthocyanins react with superoxide, nitric oxide, and peroxynitrite; these reactions induce bleaching of these compounds. The rate constant of the reaction between O_2_^−^ and cyanidin-3-sophoroside was estimated to be 2.2 × 10^5^ M^−1^ s^−1^ at pH 7.0, whereas that of the aglycon cyanidin was 10-fold higher (1.9 × 10^6^ M^−1^ s^−1^). These rate constants are considerably lower than that of copper-zinc superoxide dismutase (2.3 × 10^9^ M^−1^ s^−1^) but comparable to those of ascorbate and glutathione (GSH) (2.7 × 10^5^ M^−1^ s^−1^ and 6.7 × 10^5^ M^−1^ s^−1^, respectively) [31]. Delphinidin-3-(p-coumaroylrutinoside)-5-glucoside (nasunin) directly scavenged superoxide radicals with a potency of 143 superoxide dismutase (SOD)-equivalent units mg^–1^ [67]. Among 15 bilberry anthocyanins, the potency of activity toward the superoxide radical was in the following order: delphinidin > petunidin > malvidin ≈ cyanidin > peonidin > pelargonidin [32].

Both aglycone structure and the attached sugar moiety affected the superoxide and peroxynitrite scavenging activities, although the effect of the attached sugar moiety was smaller than that of the aglycone structure. The activity toward the superoxide radical increased in the following order: delphinidin > petunidin > malvidin ≈ cyanidin > peonidin > pelargonidin [32]. Another study showed that the EC_50_ values for superoxide scavenging were 6.96, 29.39, 31.24, 31.51, 45.94, and 207.2 µM for petunidin 3-*O*-glucoside, kuromarin, oenin, myrtillin, cyanidin 3-*O*-rutinoside, and ascorbic acid, respectively [64].

Kinetic calculations based on quantum mechanics showed that anthocyanidins are potent scavengers of superoxide (O_2_^•−^) and hydroperoxyl (HOO^•^) radicals in polar media. Twelve natural anthocyanidins were calculated to have good scavenging activity for HOO^•^ in an aqueous medium, with overall rate constants of 1.58 × 10^8^–7.59 × 10^9^ M^−1^ s^−1^. The mono- and dianionic forms of anthocyanidins, and the SET mechanism, play a dominant role in HOO^•^ scavenging by the anthocyanidins in water at a neutral pH. Superoxide scavenging of the anthocyanidins should occur rapidly in polar solvents with apparent rate constants k_app_ of about 10^9^ M^−1^ s^−1^. The O_2_^•−^ and HOO^•^ radical scavenging can occur in a regeneration cycle that might increase the protective effects of anthocyanidins against oxidative stress [68].

The EC_50_ values for nitric oxide scavenging under the experimental conditions applied of cyanidin, oenin, myrtillin, cyanidin 3-*O*-rutinoside, and petunidin-3-*O*-glucoside were 15.58, 11.81, 15.52, 18.14, and 34.17 µM, respectively, much lower compared to ascorbic acid (1432 µM) [64]. Comparison of reactivities of several anthocyanins with nitric oxide showed the following sequence of reactivities: petunidin-3-galactoside > peonidin-3-galactoside > cyanidin-3-glucoside > malvidin-3-galactoside > cyanidin-3-galactoside > cyanidin-3-arabinoside > peonidin-3-glucoside > peonidin-3-glucoside = malvidin-3-glucoside > delphinidin-3-glucoside > delphinidin-3-galactoside > delphinidin-3-arabinoside. The sequence of reactivity with peroxynitrite was as follows: peonidin-3-galactoside > petunidin-3-galactoside > cyanidin-3-glucoside > cyanidin-3-arabinoside > peonidin-3-glucoside > malvidin-3-glucoside > malvidin-3-galactoside > petunidin-3-glucoside = cyanidin-3-galactoside > delphinidin-3-arabinoside > delphinidin-3-galactoside > delphinidin-3-glucoside [69]. For anthocyanidins, the sequence of reactivity toward peroxynitrite was as follows: delphinidin > cyanidin ≈ petunidin > malvidin > peonidin > pelargonidin. Methylation of C4′-OH markedly reduced the antioxidant activity of anthocyanidins [32]. Apigeninidin showed different behavior towards different radicals with no activity towards hydroxyl and nitric oxide, but good activity towards lipids and ascorbyl radicals [70].

Anthocyanins and anthocyanidins protect proteins from nitration by peroxynitrite being themselves nitrated and degraded by peroxynitrite. In the case of pelargonidin, the main reaction products were *p*-hydroxybenzoic acid and 4-hydroxy-3-nitrobenzoic acid [37].

The efficiency of peroxyl radical trapping by various anthocyanins was related to the B ring substituents and displayed the rank order of –OH > –OCH_3_ ≫ –H as inferred from inhibition of peroxidation of linoleic acid, e.g., delphinidin 3-glucoside, which has two –OH substituents in positions C3′ and C5′, was found to be eight-fold more efficient than pelargonidin 3-glucoside which had two –H substituents on its B ring [71]. Interestingly, glycosylation at C3 and C5 of the anthocyanin skeleton have shown an enhancing effect in the chemiluminescence intensity dependent on lipid peroxidation [72]. Anthocyanins react also with hydrogen peroxide. The contribution of anthocyanidins to the scavenging of H_2_O_2_ in apple peel was reported to be greater than that of other phenolics [34].

All anthocyanins and anthocyanidins were highly antioxidative against the liposomal system and reduced the formation of malondialdehyde (MDA) by UVB (320–290 nm) irradiation. The inhibition of LDL oxidation by anthocyanins was explained by several antioxidant mechanisms including hydrogen donation, metal chelation, and protein binding [73].

Anthocyanins and anthocyanidins dose-dependently inhibited Cu^2+^-mediated LDL peroxidation. The order of efficiency in extending the lag time was as follows: cyanidin > delphinidin > cyanidin-3-glucoside > malvidin > malvidin-3-glucoside. Anthocyani(di)ns with the *o*-dihydroxy arrangement like cyanidin chelate Cu^2+^ ions, which, in part, explains their greater efficacy over the other (poly)phenols in this model oxidation system [74]. In a liposomal system, the efficiency of inhibition of lipid peroxidation increased with an increasing number of hydroxyl substituents present on the B ring. However, substitution by methoxyl groups diminished the antioxidant activity of the anthocyanidins. Substitution at position C3 of ring C played a major role in determining their efficacy in inhibiting lipid peroxidation. The anthocyanidins, which possess a hydroxyl group at position C3, demonstrated potent antioxidant activities. For the cyanidins, increasing number of glycosyl units at position C3 resulted in decreased antioxidant activity [75].

## 4. Electrochemical Properties of Anthocyanins and Anthocyanidins

Electrochemical studies are another source of data on the general trends in the electron donating abilities of anthocyanins. properties of delphinidin, cyanidin, pelargonidin, kuromanin, and callistephin were studied. The less positive potential peaks displayed by these compounds correspond to oxidations of the more redox-active OH groups of ring B. The potentials become successively more positive for oxidations of the C3-OH group and of the ring A resorcinol groups (Table 1). Delphinidin displayed four oxidation processes; the two less positive peaks correspond to C3′,C4′,C5′-OH oxidations, the third process corresponds to C3-OH oxidation, and the fourth process to C5,C7-OH oxidations. The same processes were observed for cyanidin except for the fact that the two less positive peaks corresponded to C3′,C4′-OH oxidations [63].

Differential pulse voltammetry data for anthocyanin oxidation brought values of two oxidation peaks, P1 and P2, associated with the two oxidizable centers for several anthocyanins and anthocyanidins. The values obtained at pH 7 are shown in Table 2.

Data shown in Table 2 point to the following sequence of oxidation of the hydroxyl group on C4′ of the B ring: a hydroxyl group in the pyrogallol structure (as in myrtillin) > a hydroxyl group in the *ortho* position to two methoxyl groups (as in oenin) > a hydroxyl group in the *ortho* position to a methoxyl group (as in petunidin).

A reversible oxidation reaction occurs only when a hydroxyl group on C4′ has a neighboring methoxyl group in the *ortho* position. When the hydroxyl group is located in the *ort021ho* position, next to two methoxyl groups, the reaction becomes irreversible. Glycosylation of the A ring does not affect the oxidation peak potential of hydroxyl groups on the B ring. Irreversible oxidation of the hydroxyl groups on the A ring was observed at higher positive values of the potential [76].

## 5. Effect of Interactions on the Antioxidant Activity of Anthocyanins

The absorption spectra of a cyanidin derivative showed a ca 15 nm bathochromic shift when mixed with calf thymus DNA, indicating the formation of a complex. The authors claimed that the complex formation protected both the anthocyanidin and DNA from the oxidative damage by hydroxyl radicals although no quantification of the extent of damage was performed [77]. It is obvious that in a mixture of compounds, each of them is protected by another compound acting as a competitive substrate for a radical with low substrate specificity, such as the hydroxyl radical. However, complexing may hide susceptible groups inside the complex; such a situation was postulated for the anthocyanin-DNA complex where a positively charged flavylium group binds probably to the negatively charged phosphate group of DNA [77].

Similarly, binding to proteins like Bovine Serum Albumin (BSA) protected anthocyanins against degradation and loss of antioxidant activity induced by light and ascorbate [78]. Anthocyanin stability and antioxidant activity was demonstrated to be enhanced also by interaction with whey and other proteins [79,80].

Interactions of anthocyanins with other flavonoids may affect their antioxidant activity. In the DPPH^•^ decolorization assay, interaction of anthocyanins with other flavonoids produced mainly weak antagonistic effects, although a synergistic effect was found in a mixture of kuromanin and myricetin-3-glucoside [81]. Synergistic effects of anthocyanins, hydrolyzable tannins, and/or polymeric compounds, resulting in higher radical scavenging activity of their mixtures, were also found [82].

**Figure 4:**
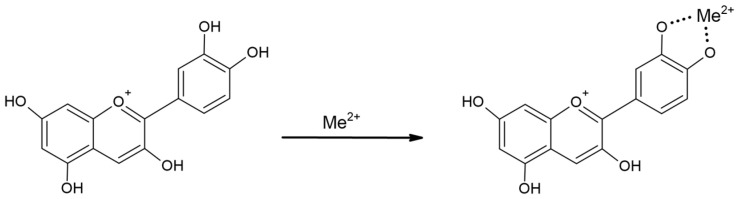
Metal binding by anthocyanidins, exemplified for cyanidin (after [43], modified).

Anthocyanins form complexes with the metal ions by using the C3- or C5-hydroxyl substituents or hydroxyl groups in the ortho position in the B ring [43] (Figure 4). This metal ion-chelating activity of anthocyanins is important for the inhibition of metal-catalyzed oxidation reactions, especially lipid peroxidation [74].

The formation of a blue color of some flowers requires interaction between the quinonoidal base with other compounds, especially metal ions. This phenomenon is referred to as co-pigmentation. Complexation with metals stabilizes the ionized quinoidal base, as the oxygen atoms involved in metal binding are no longer available for oxidation reactions, reducing the antioxidant capacity of anthocyanins and preventing their degradation in reactions with oxygen [46,83]. However, interaction with transition metal ions under in vitro conditions may lead to pro-oxidant activity of anthocyanins, similarly to other flavonoids [84]. Anthocyanins also prevented the oxidation of ascorbic acid, through chelate formation with the metal ions, and, finally, by the formation of an ascorbic acid–metal–anthocyanin complex [74].

## 6. In Vitro Cellular Antioxidant Effects of Anthocyanins

Numerous studies demonstrated antioxidant activity of anthocyanins and anthocyanidins at the cellular level, both in intact cells, and in cells challenged with various agents, simulating various pathophysiological conditions. It would be impossible to cite all of them, so, only representative studies are exemplified, classified as the most relevant by Google Scholar in a search using the combination of terms “anthocyanidin”, ”antioxidant”, and “cells” (Table 3).

Inhibition of progesterone synthesis in R2C Leydig cells subjected to AAPH-induced oxidative stress is mediated by damage to a steroidogenic acute regulatory protein (StAR), a key protein for progesterone synthesis. Anthocyanidins had a protective effect, the order of protection being cyanin > myrtillin ≈ callistephin > kuromanin. The anthocyanins decreased the level of ROS measured in cells. It was suggested that on this basis that anthocyanins may be useful in the protection against oxidative stress-induced steroidogenesis disorders [62].

## 7. Antioxidant Effects of Anthocyanins in Animal Studies

Many results have been published concerning the impact of anthocyanin-rich compounds on oxidative stress parameters in healthy animals and humans, in animals subjected to various treatments, and in patients with various diseases. Only exemplary results are shown, chosen on a similar basis as above but using a different combinations of terms (“animal” or ”human”, respectively, instead of “cells”). Chosen results of animal studies are presented in Table 4.

It has been reported that young male Eurasian blackcaps *Sylvia atricapilla* with multiple parasite infections favored anthocyanin-enriched food. This result was interpreted by oxidative stress caused hemosporidian infections, which may increase individual antioxidant needs [143]; however, this interpretation may be arbitrary.

## 8. Human Studies

Many studies on the effects of anthocyanin intake on healthy volunteers and patients suffering various diseases have been reported. Some studies did not reveal any significant effects of anthocyanin additives, but a vast majority of published papers reported amelioration of various parameters of oxidative stress by administration of anthocyanins (Table 5). Among these examples, amelioration of exercise-induced stress by anthocyanins was demonstrated. It should be considered, however, that physical exercise causes a physiological shift in the cellular redox status through an increase in ROS/RNS, which is vital for the activation of cell signaling that supports the beneficial adaptive health and ergonomic outcomes. Preventing exercise-induced changes in cell redox status is likely to inhibit/delay adaptation to exercise [144,145].

A systematic review of randomized controlled trials analyzing 28 studies involving more than a single dose and 1- to 24-week berry consumption on biomarkers of oxidative stress revealed that, of 56 oxidative stress biomarkers evaluated in the 28 RCTs, 32% of the biomarkers were reported to have statistically significant beneficial results and 68% of the biomarkers were reported as showing no statistically significant differences [162]. Meta-analysis of 21 papers on the consumption of pomegranate juice pointed to an elevation in the levels of blood plasma TAC, reduction in the level of the MDA, and no consistent changes in the levels of TAC determined by FRAP, ox-LDL, PON 1, and erythrocyte GSH and GSH-Px activity [163]. Another meta-analysis based on 33 records, confirmed elevation in the levels of blood plasma TAC, and reduction in the level of MDA [164]. Meta-analysis of randomized controlled trials on the effect of dietary anthocyanins on biomarkers of oxidative stress, based on 23 papers, showed that that the administration of dietary anthocyanins reduced the levels of MDA, Ox-LDL, and isoprostane, while increasing the level of TAC and activity of erythrocyte SOD and GPx. Compared to healthy subjects, dietary anthocyanins were more useful for unhealthy subjects [165]. A recent systematic review pointed to the effectiveness of anthocyanin-containing foods and nutraceuticals in mitigating oxidative stress associated with cardiovascular disease [166].

A meta-analysis of 39 studies observed the effect of dietary anthocyanins on exercise recovery. In most studies, anthocyanins increased TAC and decreased SOD immediately post-exercise, the most beneficial effect on the biomarkers being noted following metabolically biased exercise and longer-term interventions [167]. A meta-analysis of eight studies showed that anthocyanin intervention in patients undergoing hemodialysis decreased oxidant stress parameters, especially the MDA level [168].

The protection against effects of food-borne contaminants, including oxidative stress, by antioxidants, has been recently reviewed [169].

## 9. Discussion

### 9.1. Validity of In Vitro Cellular Experiments

While in vitro experiments are rightly recommended for the sake of the restriction of unnecessary sacrifice of experimental animals, limitations of in vitro cellular experiments should not be forgotten. Firstly, if plant extracts are studied, cells are exposed to a mixture of compounds, some of which cannot reach them in vivo because of the permeability barrier. Secondly, extensive transformation of anthocyanins to their metabolites by the gut microbiota and body’s detoxification systems may produce metabolites that are not present in the culture medium [170,171,172]. Thirdly, phenolic compounds in cell culture media, of much higher oxygen concentrations than inside the organism and near-neutral pH, are subject to autoxidation, producing, ultimately, hydrogen peroxide. This phenomenon may lead to experimental artefacts [173,174]. Thus, one should be aware that results of in vitro experiments do not always directly extrapolate on the effects which could be attained in vivo. These remarks are not meant to disavow in vitro experiments, which are invaluable element of studies of xenobiotics, but to remind individuals that they are a useful but not universal tool, like a screwdriver, allowing us to deal with screws but not with nails.

### 9.2. The Effects Are Time- and Concentration-Dependent

For obvious reasons, animal experiments, especially, are often performed employing single dose and fixed time measurements. However, the effects are dependent both on dose and time of exposure. To give an example, intragastrical administration of rabbit eye blueberry (*Vaccinium ashei* cv. ‘Brightwell’) to mice resulted in transient time-dependent increases of various parameters of blood plasma, eyeball, intestine, liver, and adipose tissue (Table 4). The measured parameters changed dynamically, sometimes decreasing below and increasing over the initial level over the time of the experiment (0.5–12 h) [116]. Fixed time measurements could lead to contradictory conclusions; moreover, the parameters may change differently in different organs. Such effects contribute to the variability of published results.

### 9.3. Direct vs. Indirect Action of Anthocyanins

What is an antioxidant? A chemical definition states that an antioxidant is a substance “which, when present at low concentrations, compared to those of an oxidizable substrate, will significantly delay or inhibit oxidation of that substrate” [175]. This definition, describing a direct action of an antioxidant, is perfect for a test tube but becomes insufficient for a culture vessel containing living cells or for a plant, animal, or human organism. In these more complex cases, an antioxidant is seen as “a substance that protects cells from the damage caused by free radicals” [176] (and other ROS/RNS). In these cases, the protective effect may be due to the direct antioxidant action (in the case of cultured cells when the antioxidant is present at high concentration), but this effect is highly unlikely to be of importance in vivo, especially for anthocyanins. These compounds are characterized by low bioavailability and reach peak concentration in the low micromolar range in body fluids when ingested [177,178,179]. Such concentrations are negligible compared to other antioxidants present inside the cells at millimolar concentrations. Encapsulation of anthocyanins may be an efficient way to circumvent this limitation, enabling supplementation with higher doses of anthocyanins and allowing for the avoidance of their jejunal degradation [180,181].

The beneficial antioxidant effects of anthocyanins at the organismal level seem to be due rather to the stimulation of the own antioxidant defense systems of the cells, as discussed by Forman et al. [182]. Indeed, many articles cited in this paper document increases, or attenuation of decreases, in the activities of antioxidant enzymes and the level of glutathione. Such indirect antioxidant effects are, in fact, due to the influence of anthocyanins on gene expression and signaling pathways and should be classified rather as signaling effects. The boosting of endogenous antioxidants and improvement of oxidative status may be mediated by various mechanism, including activation of the Nrf2 factor and anti-inflammatory action, as inflammation intensifies oxidative stress [183].

Consumption of anthocyanins has been documented to involve interference with signaling pathways, including Nrf2, leading to activation of biosynthesis of antioxidant proteins and NFκB, and inhibition of inflammation [184].

Consumption of a bolus of anthocyanin-rich beverages by healthy volunteers affected the nuclear erythroid 2-related factor-2 (Nrf2) and Nrf2-dependent gene transcription in human peripheral lymphocytes and DNA integrity, which is indicative for systemic effects [185]. The protective effect of anthocyanin extract of *Hibiscus syriacus* petals on HaCaT treated with H_2_O_2_ was associated with the activation of Nrf2 [90]. Pretreatment with cyanidin-3-*O*-glucoside in human umbilical vein endothelial cells activated the Nrf2/ARE pathway at baseline and after TNF-α treatment [186]. Malvidin protecting WI-38 against H_2_O_2_ prevented the increase in the NFκB protein [94]. Beneficial effects of anthocyanins in neurodegenerative diseases, involving attenuation of oxidative stress accompanying these diseases and perhaps contributing to their mechanisms, also involves interference with signaling pathways including Nrf2 and NFκB [184], and inhibition of inflammation. Modulation of oxidative stress by cyanidin-3-glucoside, as mediated by Nrf2 pathway remains, has been reviewed [187].

Another important factor which may mediate the effect of anthocyanins on the oxidative stress is the effect on the intestinal microbiome [188].

### 9.4. How Specific Are the Anthocyanin Effects?

It should be taken into account that anthocyanins are subject to metabolic transformations and degradation, i.a., by the intestinal microbiome. Intestinal microbiome degrades anthocyanins to protocatechuic acid, gallic acid, syringic acid, vanillic acid, ferulic acid, 4-hydroxybenzoic acid), hippuric acid, phloroglucinol carboxaldehyde, coumaric acid, and 2-hydroxy-4-methoxybenzoic acid [189,190]. Therefore, many effects attributed to anthocyanins may be, in fact, due to the action of their metabolites or degradation products [191,192,193,194], though it is of secondary importance for recommendations concerning anthocyanin intake.

Consumption of food rich in anthocyanins was reported to exert a number of antioxidant effects on the whole-body level. These effects are attributed to anthocyanins but it should be kept in mind that, unless pure anthocyanin extract was used, other antioxidants present in food might have contributed to these effects, e.g., anthocyanin-rich cranberry juice was found to contain 2.80 mg anthocyanins (malvidin-3-glycoside equivalents)/L but also 29.06 mg catechins/L and 897 mg vitamin C/L [146] The pomegranate juice contained 1979 mg/L of tannins, 384 mg/L of anthocyanins, 121 mg/L of ellagic acids derivatives, and 30 mg/L of vitamin C [158]. The “anthocyanin-rich fruit juice” produced by Eckes-Granini GmbH (Niederolm, Germany) from red grape juice, lingonberry juice, apple, blueberry, strawberry, and aronia juice, and acerola puree contained 274.5 mg anthocyanins/L, 3.6 g total polyphenols/L, and 564 mg vitamin C/L. Effects caused by administration of this juice to healthy volunteers (750 mL/day for up to 8 weeks), including reduction in blood DNA strand breaks, were similar in persons receiving the juice and in the groups receiving placebo, which was lacking anthocyanins but contained a somewhat higher amount of vitamin C [195]. In another study, the pomegranate juice used contained 384 mg/L of anthocyanins, 1979 mg/L of tannins, 121 mg/L of ellagic acid derivatives, and 30 mg/L of vitamin C [161]. The total anthocyanin content of blackberry juice contained about 312.9 mg cyanidin 3-glucoside/100 g DW while the total flavonoid content was about 36.7 mg QE/g DW, and total polyphenol concentration of about 28.80 mg gallic acid equivalents/g DW [126]. *Moro* (a blood orange) juice contained 433.5 mg/L ascorbic acid, 80.71 mg/L anthocyanins, 201.17 mg/L flavanones, and 81.01 mg/L cinnamic acids [88]. Daily urinary excretion of phenolic compounds after consumption of 500 mg daily of aronia berry extract included 0.332 mg anthocyanins/mg creatinine and 12.98 phenolic acids/mg creatinine [151]. Considering that the antioxidant activity of various polyphenols is of the same order of magnitude, a possibility exists that the effects of anthocyanin-rich plant extracts may be contributed by, or even mainly due to, other components of the extract other than anthocyanins.

An appropriate control would be an extract of identical composition but devoid of anthocyanins. Comparison of strains producing and devoid of anthocyanins could be the right way although the content of other antioxidants can be altered in plants not producing anthocyanins. Comparison of extracts of bran of two isogenic wheat lines, one containing anthocyanin and another, anthocyanin-free showed that the anthocyanin-containing extract had higher antioxidant activity in the tests of ABTS^•^ decolorization and DPPH^•^ decolorization, and inhibition of AAPH-induced hemolysis. Both effects (0.1 g/mL) prolonged the life span of *Drosophila melanogaster* females, but the effect was stronger for the anthocyanin-free extract [196].

### 9.5. Some Remarks on Biomarkers Evaluating Antioxidant Effects

Quite a range of biomarkers are used to evaluate the effects of anthocyanins (and other antioxidants) on cells and organisms. In cellular experiments, the level of intracellular ROS, easily estimated with fluorogenic probes, is often used as a direct measure of oxidative stress. Results of such experiments are referred to in different ways, as “ROS level” or “ROS generation”. The first term seems more reasonable as “generation” should be expressed as the amount of ROS generated per unit of time. In fact what is measured is something intermediate between the level and the production because not an amount in a given moment, but this amount plus the amount of ROS generated during incubation with the probe, is measured. Moreover, the result of the measurements reflects the amount of ROS reacting with the probe; it should be kept in mind that a fraction of ROS is decomposed or scavenged by antioxidants present in a sample and cannot react with the fluorogenic probe [197]. 2′,7′-Dichlorodihydrofluorescein (H_2_DCF) was reported to be oxidized directly by heme, hemoglobin, myoglobin, and cytochrome c, without the intermediacy of ROS [198]; changes in the levels of these hemoproteins may thus affect the results.

It is of interest to note that in studies demonstrating the indirect antioxidant protective effects of anthocyanins on cells or experimental animals subjected to different treatments, anthocyanins usually attenuate the decreases in the values’ various parameters like the glutathione level. However, sometimes attenuation of an increase is reported. Are they artefacts? Both situations can occur. Often, a noxious agent depletes cells of glutathione but then a compensative increase in GSH synthesis occurs and an overcompensation is observed [199,200]. Thus, an attenuation of both a decrease and an increase in a parameter in question can be valid proof of an indirect antioxidant action. However, there are exceptions. Changes in the activities of ROS-decomposing enzymes in isolated erythrocytes, reported in some papers, most probably represent experimental artefacts, as it is hard to expect significant increases in the activities of enzymes in cells devoid of protein synthesis.

From among parameters used to evaluate stress on the organismal level, the levels of products of lipid, protein, and DNA oxidation, TAC of blood plasma(serum), and activities of antioxidant enzymes are the most often measured. Products of lipid peroxidation are most commonly measured in the reaction with thiobarbituric acid, and referred to as TBARS, but sometimes as MDA, although it is not the only product of the reaction. Sometimes the MDA content is measured using more specific methods like HPLC. The thiobarbituric acid reaction has been criticized due to its low specificity and possibilities of interference [201,202], and the more expensive analysis F_2_ isoprostanes was recommended instead [203,204].

The reported increases in the total antioxidant capacity of blood plasma following ingestion of anthocyanins are intriguing, as their magnitude considerably exceeds values which could be expected from enrichment by anthocyanins. It was reported, however, that such an effect following consumption of exogenous antioxidants can be indirect and caused by an increase in the concentration of endogenous antioxidants in the plasma [205].

## 10. Conclusions

In summary, anthocyanins are good antioxidants and attenuate oxidative stress at the cellular and organismal levels. However, considering their low bioavailability and metabolic transformations, it should be considered that their organismal effects are mainly of indirect nature, mediated by activation of their own antioxidant systems of the body, and, in many cases, may be mediated by their metabolites. Nevertheless, irrespective of the mechanism of action, the antioxidant and other beneficial effects of anthocyanins, dependent on or independent of their antioxidant properties, fully justify the recommendations of everyday consumption of food products rich in these compounds.

## Figures and Tables

**Table 1:** Values of the anodic peak potentials (E_pa_) in cyclic voltammograms of several anthocyanidins and anthocyanins. Concentration: 0.1 mM, solvent: methanol + 0.1 M lithium perchlorate. Data taken from [63].

Compound	E_pa_ vs. Ag/AgCl [mV]
Delphinidin	519, 608, 844, 1108
Cyanidin	564, 646, 1084
Pelargonidin	598, 1121
Kuromanin	678, 773, 940, 1115
Callistephin	948, 1086

**Table 2:** Values of potentials of two oxidation peaks, E_P1_ and E_P2_, derived from differential pulse voltammetry measurements at pH 7. Data from [76].

Compound	E_P1_ [mV]	E_P2_ [mV]
Myrtillin	195	490
Peonidin-3-*O*-glucoside	210	600
Petunidin	285	630
Kuromanin	310	500
Cyanin	310	630
Oenin	380	500
Malvin	380	640

**Table 3:** Chosen in vitro cellular effects of anthocyanidins.

Anthocyanin Preparation	Cells	Effect	Reference
Anthocyanin fraction of the pomegranate (*Punica granatum*) juice	Human hepatocellular carcinoma HepG2 cells	Decrease in ROS level	[82]
Anthocyanins of red clover (*Trifolium pratense*) flowers	Mouse RAW 264.7 monocytes	Decrease in ROS level	[85]
Anthocyanin-rich black rice extract or its main components (kuromanin and peonidin-3-*O*-glucoside)	HepG2 cells	Decrease in ROS level, increased SOD and CAT activities	[86]
Malvidin, oenin, and malvidin-3-galactoside	Human umbilical vein endothelial cells (HUVECs)	Decreased ROS and XO-1 levels, increased SOD level	[87]
Anthocyanin-rich *Moro* (red) orange extract or cyanidin-3-glucoside	Mouse adipocytes	Decreased basic and insulin-induced ROS level	[88]
Anthocyanin extract from the skin of grape berries	Human retinal pigment epithelial cells ARPR-19 treated with H_2_O_2_	Increased SOD, CAT, GPx, and GST activities, decreased lipid peroxidation	[89]
Anthocyanin extract of *Hibiscus syriacus* petals	Human immortalized keratinocytes HaCaT treated with H_2_O_2_	Protection against apoptosis and increase in ROS level	[90]
Anthocyanin extract of *Rubus* blackberries fruits	RAW 264.7 cells treated with H_2_O_2_	Decreased ROS level, increased SOD and GPx activities, reduced DNA oxidation	[91]
Anthocyanin-rich hydroethanolic extract of pigeon pea (*Cajanus cajan*) and its major component, kuromanin	RAW 264.7 cells treated with H_2_O_2_	Protection against DNA damage	[92]
Blueberry anthocyanin extract, malvidin, myrtillin, and malvidin-3-galactoside	Human retinal pigment epithelial cells	Decreased ROS and MDA levels, increased SOD, CAT, and GPx levels	[93]
Malvidin	Human WI-38 fibroblasts treated with H_2_O_2_	Amelioration of increases in the levels of MDA, and COX-2 and NFκB proteins	[94]
Black rice anthocyanin extract	Rat pheochromocytoma PC-12 cells treated with H_2_O_2_	Increased survival, smaller decreases in SOD and CAT activities, attenuation of increase in MDA levels	[95]
Anthocyanin extract of sweet cherries (*Prunus avium*)	Human erythrocytes exposed to AAPH	Protection against hemolysis and oxidation of hemoglobin	[96]
Blackberry anthocyanin extract	Human colorectal carcinoma Caco-2 cells exposed to AAPH	Reduced oxidation of intracellular DCFH_2_	[97]
Anthocyanin-enriched extract of the lemon bottle-brush *Callistemon citrinus* flowers	Human erythrocytes exposed to AAPH	Amelioration of increase in TBARS content, hemoglobin oxidation, decrease in GSH and total thiol content, band 3 phosphorylation, increases in SOD and CAT activities	[98]
Oenin	Bovine aortic endothelial cells treated with peroxynitrite	Attenuation of protein carbonyl formation	[99]
Kuromanin, myrtillin > callistephin	Bovine aortic endothelial cells treated with peroxynitrite	Decrease in the level of induced ROS	[100]
Black carrot anthocyanins	Human neuroblastoma SH-SY5Y cells treated with MPTP	Attenuation of increase in ROS level	[101]
Blueberry anthocyanin extract, malvidin, oenin, malvidin-3-galactoside	HUVECs exposed to high glucose concentration	Increased SOD and heme oxygenase-1 levels, lowered ROS level	[102]
Anthocyanin extract of blueberries and its main components (malvidin, malvidin-3-glucoside, and malvidin-3-galactoside)	Human retinal capillary endothelial cells exposed to high glucose concentration	Attenuation of increase in ROS level and decreases in SOD and CAT activities	[103]
Anthocyanin extract of *Aronia melanocarpa* fruits	Mouse TC3 pancreatic β cells treated with high glucose concentrations	Increase in CAT and GPx activities	[104]
Anthocyanin extract of *Aronia melanocarpa* fruits	Mouse TC3 pancreatic β cells treated with H_2_O_2_	Increase in GPx activity	[104]
Anthocyanin extract of *Aronia melanocarpa* fruits	Mouse TC3 pancreatic β cells treated with high glucose concentrations or H_2_O_2_	Attenuation of decrease in the GSH content	[104]
Cyanidin-3-glucoside	ARPE-19 cells exposed to high glucose concentration	Attenuation of increase in ROS level	[105]
Anthocyanin-rich sour cherry extract	HUVECs exposed to high glucose concentration	Amelioration of increase ROS level and of decrease in eNOS expression	[106]
Anthocyanin-rich mulberry extract	HUVECs treated with oxidized LDL	Decreased superoxide production and nitrotyrosine level, increased NO release	[107]
Malvidin	RAW 264.7 cells stimulated by LPS	Decreased ROS production	[108]
Anthocyanin extracted from black soybean seed coats	RAW 264.7 cells stimulated by LPS	Decreased ROS production	[109]

AAPH, 2,2′-azobis(2-amidinopropane); CAT, catalase; COX, cyclooxygenase; DCFH_2_, 2′,7′-dichlorodihydrofluorescein; eNOS, endothelial nitric oxide synthase; GSH, glutathione; GPx, glutathione peroxidase; GST, glutathione *S*-transferase; LPS, bacterial lipopolysaccharide; MDA, malondialdehyde; MPTP, 1-methyl-4-phenyl-1,2,3,6-tetrahydropyridine; ROS, reactive oxygen species; SOD, superoxide dismutase; TBARS, thiobarbituric-acid reactive substances; XO, xanthine oxidase.

**Table 4:** Chosen effects of anthocyanidins in animal experiments.

Anthocyanin Preparation	Animals Studied	Effect	Reference
Black rice anthocyanin extract (70–280 µg/mL)	*Cenorhabditis elegans*	Lifespan prolongation, increased resistance to oxidative stress, increased activities of SOD and CAT, reduced lipofuscin accumulation, ROS, and MDA levels	[95]
Kuromanin (25 µM)	*Caenorhabditis elegans* exposed to 100 µM H_2_O_2_	Lifespan prolongation	[110]
Anthocyanin extracted from coffee (*Coffea arabica*) husks, both fresh (50 µM) and stabilized in ZnO nanoparticles	*Caenorhabditis elegans* exposed to H_2_O_2_	Increased survival	[111]
Bilberry anthocyanin extract	Aging female rats	Increased TAC, total SOD, and CAT activities, decreased MDA level in serum	[112]
Anthocyanin-rich red potato flakes	Rats	Increased blood serum TAC, decreased TBARS level in blood serum and liver, decreased expression of genes coding for MnSOD and CuZnSOD in the liver	[113]
Blackberry extract enriched in anthocyanins (35 d)	Rats	Increased blood plasma TAC, reduced liver TBARS level, increased liver, kidney, and brain GSH levels, increased erythrocyte and kidney GPx activities, increased liver SOD activity, increased CuZnSOD and MnSOD gene expression in the kidneys, and GPx gene expression in the spleen	[114]
Enrichment of rat diet with freeze-dried fruit of cornelian cherry *Cornus mas* (5 wk)	Rats	Increased brain and serum CAT and PON 1 activities	[115]
Rabbit eye blueberry (*Vaccinium ashei* cv. ‘Brightwell’) (100–800 mg/kg bw), administered intragastrically	Mice	Transient increases of blood plasma TAC, eyeball, intestine, liver, and adipose tissue. SOD and GPx activities. Decreased MDA content	[116]
Injection of anthocyanin (10–30 ppm)	Eggs containing developing embryos of broiler chicks	Increased hatchability, and GPx activity, decreased MDA content	[117]
Anthocyanin extracts from blackberry (*Rubus* sp.) and blueberry (*Vaccinium ashei*)	Mice fed high-fat diet	Prevention of increase in hepatic MDA and of decreases in activities of hepatic SOD and GPx	[118]
Anthocyanin-rich mix (NSE Products, Inc. Provo, UT, USA) (40 mg AC/kg bw, 14 w)	Mice fed high-fat diet	Mitigation of upregulation of NOX1, NOX4, and NOS2, and of increase in the level of HNE-protein adducts	[119]
Anthocyanin-rich mulberry extract	Rats fed high-fat diet	Attenuation of increase in the levels of oxidized LDL in serum, superoxide, and MDA adducts in aorta	[107]
Pomegranate byproduct (whole pomegranate fruit left after juice preparation)	Apolipoprotein E-deficient (E°) mice	Reduced lipid peroxide content, attenuation of increase of GSH content in peritoneal macrophages, increased PON 2 activity	[120]
Cyanidin-3-*O*-β-glucoside (2 g/kg diet, 8 w)	ApoE-deficient mice with hypercholesterolemia-induced endothelial dysfunction	Decreased levels of ketosterol, superoxide, and lipid peroxides in the aorta	[121]
Anthocyanin-containing alcoholic extract of *Amaranthus caudatus* (150 mg of lyophilized extract/(kg bw × d))	Rabbits fed hypercholesterolemic diet	Regression of atherosclerotic lesions, without reduction in serum levels of MDA and oxidized LDL	[122]
Dried chokeberry (*Aronia melanocarpa*)	Mice treated with D-galactose	Prevention of increase in serum, kidney, and liver (but not brain) MDA levels	[123]
Anthocyanin-rich pomegranate juice sugar fraction (10 d)	Diabetic mice	Decreased ROS level, increased GSH level in macrophages; opposite effects of analogous fraction isolated from white grapes	[124]
Anthocyanin * (100 mg/kg bw, 8 wk)	Rats with streptozotocin-induced diabetes	Attenuation of increase in the MDA level, and decreases in the SOD and GPx activities in serum	[125]
Anthocyanin-rich blackberry juice (9 mL/kg bw, 56 d)	Rats with streptozotocin-induced diabetes	Amelioration of decreases in SOD, CAT, and GPx activities and of increase in liver MDA content	[126]
Blueberry anthocyanin extract (20–80 mg/kg bw), 10 wk	Mice with streptozotocin-induced diabetes	Alleviation of decrease in TAC and SOD activity and of increase in MDA content in the retina	[127]
Black bean peel anthocyanin extract (200 or 400 mg/kg bw, 4 wk)	Rats with streptozotocin-induced diabetes	Amelioration of decreases in SOD and CAT activities and of increases in MDA level in serum	[128]
Anthocyanin extract of purple sweet potato (*Ipomoea batatas*) (50 and 100 mg/kg bw, 35 d)	Rats with alloxan-induced diabetes	Amelioration of increase in MDA levels in blood, liver, and kidneys	[129]
Cyanidin-3-*O*-β-glucoside (200 or 400 mg per kg bw, 4 wk)	Rats with monocrotaline-induced lung artery hypertension	Reduction in MDA level increase and SOD activity decrease in plasma	[130]
Purple sweet potato extract (0.42 mg of anthocyanins in 100 g diet)	Rats fed high-fat diet inducing a model of dry eye disease	Mitigation of the drop in CuZnSOD level in the lacrimal gland	[131]
Malvidin (100 mg/kg bw, 21 d)	Rats with ischemia-reperfusion kidney injury	Amelioration of increase in renal MDA level and of decreases in SOD and CAT activities	[132]
Anthocyanin extract from purple sweet potatoes (10 mg/kg bw, 2 wk)	Mice subjected to restraint stress	Decreased in MDA content, prevention of increase in SOD activity in the liver	[133]
Anthocyanin * (100 mg/kg bw, administered 30 min before the experiment)	Rats subjected to ovary torsion/detorsion	Amelioration of decreases in serum SOD and GPx activities and of increase in serum MDA level	[134]
Ethanolic (61%) extract of bilberry (100 or 400 mg AC/kg bw, 21 d)	Female broiler chicks treated with LPS	Mitigation of loss of plasma jejunum mucosa and liver SOD activities, and of increase in the MDA content in jejunum mucosa and liver	[135]
Anthocyanins purified from winged yam *Dioscorea alata* (10 mg/kg bw, 3 d)	Mice treated with LPS	Amelioration of decreases in serum TAC, SOD, and GPx activities and of increase in serum MDA level	[136]
Blueberry (*Vaccinium uliginosum*) anthocyanins extract (35 or 175 mg/(kg bw × d), 19 d)	Rats intoxicated with acrylamide	Amelioration of decreases in SOD, CAT, and GPx activities and GSH level, and of increase in the MDA level in hippocampus, cortex, and blood serum	[137]
Russian box thorn (*Lycium ruthenicum*) anthocyanin extract (500 mg/(kg bw × d)	Rats intoxicated with CdCl_2_	Prevented of cadmium-induced increase in serum ROS level and testicular MDA content, and of decreases in SOD, CAT, and GR activities and GSH content	[138]
Blueberry anthocyanins extract	Mice intoxicated with NaAsO_2_	Amelioration of decreases in SOD and CAT activities and TAC, and of increase in MDA level in serum	[139]
Extract of air-dried fruit of cornelian cherry *Cornus mas* (300 and 700 mg/kg bw, 16 d)	Rats intoxicated with CCl_4_	Mitigation of decreases in SOD, CAT, and GPx activities and of increase in MDA content in the liver	[140]
Extract of air-dried fruit of cornelian cherry *Cornus mas* (200 and 500 mg/kg bw, 16 d)	Rats intoxicated with CCl_4_	Mitigation of decreases in SOD, CAT, and GPx activities and of increase in MDA content in the liver	[141]
Extract of air-dried fruit of cornelian cherry *Cornus mas* (300 and 700 mg/kg bw, 16 d)	Rats intoxicated with CCl_4_	Mitigation of decreases in SOD, CAT, and GPx activities and of increase in MDA content in the kidneys	[142]

AC, anthocyanin; bw, body weight; GR, glutathione reductase; HNE, 4-hydroxynonenal; NOS, nitric oxide synthase; NOX, NADPH oxidase; PON, paraoxonase; TAC, total antioxidant capacity; * details not reported.

**Table 5:** Examples of human intervention studies employing anthocyanins.

Anthocyanin Preparation	Subjects Studied	Effect	Reference
Cranberry juice (750 mL/day, 2 wk)	Healthy volunteers	No significant effect on blood plasma TAC and MDA level, 8-OHdG excretion in urine and 8-OHdG in lymphocyte DNA	[146]
Anthocyanin-rich purple-flesh potatoes (150 g/d of cooked potato, 6 wk)	Healthy volunteers	No effect on blood plasma TBARS and protein carbonyl levels, lower plasma 8-OHdG concentration (with respect to volunteers eating white potatoes)	[147]
Purple-flesh potatoes (consumption of 300 g)	Healthy volunteers	A 160% higher plasma TAC 6 h after consumption	[147]
Anthocyanin-rich açaí (*Euterpe oleracea*) juice (200 mL/day, 4 wk)	Healthy adults	Increased blood serum and erythrocyte CAT and GPx activities in healthy adults	[148]
Anthocyanin-rich lyophilized grape powder extract (36 g/d, 4 wk)	Premenopausal and postmenopausal women	Decreased urinary excretion of prostaglandins	[149]
Tart cherry juice (240 mL twice daily, 14 d)	Elderly persons (61–75 y)	Reduced basal urinary excretion of 8-OHdG, enhanced capacity to resist oxidative damage in response to forearm I/R as evaluated by changes in plasma F_2_-isoprostane level	[150]
Aronia berry extract (500 mg daily, 4 wk)	Former smokers	No effect markers on urinary 8-isoprostane excretion and ox-LDL level in plasma	[151]
Maqui berry (*Aristotelia chilensis*) extract, Delphinol^®^ (162 mg ACs, three times daily for 4 weeks)	Healthy adults, overweight adults, and adult smokers	Reduced level of ox-LDL, decrease in urinary F_2_-isoprostanes (8-iso-prostaglandin F2α) excretion	[152]
New Zealand blackcurrant anthocyanin-rich extract) (3.2 mg/kg bw, 1 h before experiment)	Volunteers subjected to a 30-min rowing exercise	Attenuation of the transient increase in the level of plasma protein carbonyls	[153]
Antioxidant supplement containing black grape, raspberry, and red currant concentrates, 30 min before experiment)	Volunteers subjected to a 90-min bicycle ergometric exercise	Attenuation of the exercise-induced increase in the levels of plasma protein carbonyls and urinary 8-OHdG	[154]
Freeze-dried whole blackcurrant fruit extract (48 g of blackcurrants, half before and half after the experiment)	Volunteers subjected to a 30-min rowing exercise	Attenuation of the exercise-induced increase in plasma protein carbonyls	[155]
Anthocyanin-rich New Zealand blackcurrants (3.2 mg ACs/kg bw, 5 wk)	Volunteers subjected to a 30-min rowing exercise	Attenuation of the exercise-induced increase in plasma MDA level, no effect on plasma TAC	[156]
Anthocyanin-rich *Aronia melanocarpa* extract (100 mg, three times daily, 2 m)	Patients with metabolic syndrome	Increase in SOD and GPx activities, decrease in TBARS content in erythrocytes	[157]
Pomegranate juice (50 mL per day, 3 m)	Patients with non-insulin dependent diabetes mellitus	Increase in blood plasma -SH level, decrease in level of TBARS in serum, normalization of PON 1 activity and GSH level in monocyte-derived macrophages	[158]
Wild blueberry (*Vaccinium angustifolium*) freeze-dried powder (375 mg of ACs/d), 6 wk	Persons with cardiovascular risk factors	Decrease in the level of formamidopyrimidine-sensitive sites in DNA in blood mononuclear cells, decreased H_2_O_2_-induced DNA damage	[159]
Anthocyanin capsules (Medox) (320 mg ACs/d, 12 wk),	Patients with dyslipidemia	Decrease in urinary 8-OHdG and 8-iso-PG_F2α_ excretion and serum MDA level	[160]
Pomegranate juice (50 mL/d, up to 3 y)	Patients with carotid artery stenosis	Progressive decrease in the peroxide content of LDL and increase in serum PON 1; decrease in the content of lipid peroxides and increase in the GSH content in carotid lesions	[161]

8-OHdG, 8-hydroxy-2′-deoxyguanosine; I/R, ischemia/reperfusion; ox-LDL, oxidized low-density lipoprotein; PG, prostaglandin. The effects of anthocyanins on oxidative stress parameters in healthy persons and in diseases have been the subject of reviews and meta-analyses.

## Data Availability

Data will be available upon reasonable request.
